# Prognostic Value of an Immunohistochemical Signature in Patients With Bladder Cancer Undergoing Radical Cystectomy

**DOI:** 10.3389/fonc.2021.641385

**Published:** 2021-03-25

**Authors:** Jie Wu, Jun-Miao Wen, Yu-Chen Wang, Wen-Jie Luo, Qi-Feng Wang, Hong Lv, Bo Dai, Ding-Wei Ye, Heng-Chuan Su, Yi-Ping Zhu

**Affiliations:** ^1^ Department of Urology, Fudan University Shanghai Cancer Center, Shanghai, China; ^2^ Department of Oncology, Shanghai Medical College, Fudan University, Shanghai, China; ^3^ Department of Radiation Oncology, Fudan University Shanghai Cancer Center, Shanghai, China; ^4^ Department of Pathology, Fudan University Shanghai Cancer Center, Shanghai, China

**Keywords:** bladder cancer, immunohistochemistry, least absolute shrinkage and selection operator, prognosis, signature

## Abstract

**Background:**

This study aimed to assess the prognostic value of various diagnostic immunohistochemical (IHC) markers and develop an IHC-based classifier to predict the disease-free survival (DFS) of patients with bladder cancer undergoing radical cystectomy.

**Methods:**

IHC was performed on tumor specimens from 366 patients with transitional cell bladder cancer. The least absolute shrinkage and selection operator (LASSO) Cox regression model was used to develop a multi-marker classifier for predicting DFS of patients with bladder cancer. The Kaplan–Meier estimate was performed to assess DFS, and unadjusted and adjusted Cox regression models were used to identify independent risk factors to predict DFS of patients with bladder cancer.

**Results:**

Based on the LASSO Cox regression model, nine prognostic markers were identified in the training cohort. Patients were stratified into low- and high-risk groups using the IHC-based classifier. In the training cohort, the 10-year DFS was significantly better in low-risk patients (71%) compared with high-risk patients (18%) (p < 0.001); in the validation cohort, the 10-year DFS was 86% for the low-risk group and 20% for the high-risk group (p < 0.001). Multivariable Cox regression analyses showed that the high-risk group based on the classifier was associated with poorer DFS adjusted by clinicopathological characteristics. Finally, a nomogram comprising the classifier and clinicopathological factors was developed for clinical application.

**Conclusion:**

The nine-IHC-based classifier is a reliable prognostic tool, which can eventually guide clinical decision making regarding treatment strategy and follow-up scheduling of bladder cancer.

## Background

Bladder cancer is currently the 10th most commonly diagnosed malignancy worldwide, accounting for 549,393 new cases and 199,922 deaths in 2018 (1–3). Urothelial cell carcinoma is the predominant histologic subtype of bladder cancer, contributing to more than 90% of bladder cancer cases (4). Approximately, 70% of patients are diagnosed with non-muscle-invasive bladder cancer (NMIBC), whereas the remaining have muscle-invasive bladder cancer (MIBC). For MIBC, radical cystectomy is considered as the standard treatment choice; neoadjuvant chemotherapy is also used in MIBC to improve the survival of patients (5). However, despite the aggressive treatment strategy, the 5-year overall survival (OS) rate for MIBC is approximately 50% (6, 7). Thus, clinicopathological features might not be sufficient to predict prognosis and identify patients with a high risk of disease progression. There still exist undefined molecular mechanism that promotes the tumorigenesis and progression of bladder cancer.

Immunohistochemistry (IHC) is currently the most widely used pathological technique in the accurate diagnosis of urinary bladder neoplasms (8). IHC analysis is routinely applied to determine the expression of specific cancer -associated molecules involved in several biological pathways. The representative markers comprise oncogenes (HER2, EGFR, VEGF, and CyclinD1), tumor proliferation markers (BAX, BCL2, and Ki67), multidrug resistance (MDR) gene, tumor suppressor genes (p53 and p27), and enzymes (GSTπ and TOPOII) (9–11). The identification and validation of the prognostic IHC signature have been reported in various cancer types and proved to be a promising complement in therapeutic planning and patient management (12, 13). Also, a large number of IHC markers have been used in predicting the prognosis of bladder cancer so far, but none of them have eventually entered routine clinical practice (14–16).

This retrospective study assessed the prognostic value of various IHC markers representative of different biological pathways and developed a nine-IHC-based classifier to predict the disease-free survival (DFS) of patients with bladder cancer undergoing radical cystectomy.

## Methods

### Patients and Clinicopathological Information

A total of 366 consecutive patients with bladder cancer undergoing radical cystectomy (from January 2008 to December 2015) were recruited from the Department of Urology, Fudan University Shanghai Cancer Center (FUSCC). The clinical and pathological data of each patient were reviewed and recorded, including age at surgery, sex, histology (urothelial cell carcinoma only), depth of tumor invasion (T stage), lymph node metastasis (N stage), grade, vascular invasion, perineural invasion, surgical margin status, and tumor size (**Table 1**). Patients with incomplete follow-up information, presenting variant histology, or having distant metastasis at diagnosis were excluded. Tissue samples were collected during surgery and preserved in the FUSCC tissue bank. The DFS of patients was calculated from the initiation of surgery until the first recurrence, or first progression, including metastasis or death. To develop and validate the classifier, patients were further randomly stratified into training cohort (n = 256) and validation cohort (n = 110) in a ratio of 7:3. This study was approved by the institutional ethics committee of Fudan University Shanghai Cancer Center and written informed consent was obtained from all the patients preoperatively.

**Table 1:** Demographic and clinical characteristics of patients in discovery and validation cohort.

	Training cohort (n=256)	Validation cohort (n=110)	*p* value
Gender			0.649
Male	222 (86.7)	98 (89.1)	
Female	34 (12.3)	12 (10.9)	
Age (year)			0.990
(Mean, SD)	59.36 (9.91)	59.35 (10.21)	
Depth of invasion (T stage)			0.744
Tis	4 (1.6)	2 (1.8)	
Ta	14 (5.5)	2 (1.8)	
T1	58 (22.7)	27 (24.5)	
T2	70 (27.3)	33 (30.0)	
T3	74 (28.9)	31 (28.2)	
T4	36 (14.1)	15 (13.6)	
Lymph node metastasis (N stage)			0.416
Negative	198 (77.3)	80 (72.7)	
Positive	58 (22.7)	30 (27.3)	
Grade			0.830
Low grade	22 (8.6)	8 (7.3)	
High grade	234 (91.4)	102 (92.7)	
Vascular invasion			0.894
Absent	178 (69.5)	75 (68.2)	
Present	78 (30.5)	35 (31.8)	
Perineural invasion			0.779
Absent	196 (76.6)	82 (74.5)	
Present	60 (23.4)	28 (25.5)	
Surgical margin status			1.000
Negative	232 (90.6)	100 (90.9)	
Positive	24 (9.4)	10 (9.1)	
Tumor size (cm)			0.401
(Mean, SD)	3.74 (1.86)	3.93 (2.03)	

### Immunohistochemistry

All bladder cancer tissues were collected from the overall patient cohort, fixed in 10% buffered formalin, and embedded in paraffin. Immunohistochemistry was then performed by the Immunohistochemistry Diagnostic Laboratory of FUSCC to detect the expression of diagnostic biomarkers of bladder cancer (**Supplementary Table 1**). Briefly, the sections were deparaffinized in xylene and rehydrated in graded alcohol washes. Antigen retrieval was performed in citric acid (10 mM, pH 6.0) at 95°C for 30 min (HER2, EGFR, BAX, BCL2, MDR, and GSTπ), or a Tris-based buffer (pH 8.3) solution at 95°C for 60 min (VEGF, CyclinD1, Ki67, p53, p27, and TOPOII) with the help of a microwave. The sections were then treated with 0.3% hydrogen peroxide for 30 min to block endogenous peroxide activity. Next, the slides were incubated with primary antibodies at 4°C overnight and then incubated with biotinylated anti- rabbit or anti-mouse IgG secondary antibodies (EnVision Plus; Dako, CA, USA) for 30 min at 37°C. Finally, the sections were stained using a DAB kit (Dako, Agilent Technologists, CA, USA) and counterstained with hematoxylin.

### Evaluation of Immunohistochemistry

Immunostaining reactivity was observed by two experienced pathologists blinded to the clinical features independently. The proportion of positively stained cells and the maximum intensity of IHC signal were estimated. The staining score of the surface membrane, cytoplasm, or nucleus of tumor cells was calculated based on the four-point system: IHC0 (negative), IHC1+ (weak), IHC2+ (moderate), and IHC3+ (strong). Notably, the protein expression of Ki67 was scored based on the percentage of positively stained cells in 200 cancer cells, and IHC staining of HER2 was estimated based on a gastric cancer scoring system established by Park et al. (17).

### Statistical Analysis

Demographic characteristics were summarized as counts and percentages for categorical variables. Pearson’s chi-square test was performed to analyze the distribution of categorical data. The least absolute shrinkage and selection operator (LASSO) Cox regression model was used to develop the multi-marker classifier for predicting DFS of patients with bladder cancer in the training cohort. Survival analysis was performed using the Kaplan–Meier method with p values determined by the log-rank test. Unadjusted and adjusted Cox regression models were used to identify the independent risk factors to predict the DFS of patients with bladder cancer. Factors with a p value <0.1 in the unadjusted analysis were subjected to adjusted analysis. Receiver operating characteristic (ROC) analysis was used to assess the prognostic performance of the classifier. A nomogram was developed based on the independent prognostic factors according to the multivariate Cox regression analysis. Calibration curves were employed to compare the nomogram-predicted survival probabilities and the actual survival probabilities.

All statistical assessments were evaluated at a two-sided p value of 0.05. All analyses were conducted using R software 3.5.2 (R Foundation for Statistical Computing, Vienna, Austria). The “maxstat” package was used to determine the cut-off values for continuous variables. The “glmnet” package was used to conduct the LASSO Cox regression model analysis. The “survival” and “survminer” packages were used to perform survival analysis. The “timeROC” package was used to plot the ROC curves and determine the area under the curve (AUC). The “rms” package was used to develop nomogram and calibration curves.

## Results

### Demographic and Clinical Characteristics of Patients

The demographic and clinical characteristics of 366 patients with bladder cancer undergoing radical cystectomy are presented in **Table 1**. The mean age of the patients was 59.36 years (9.98), and the median follow-up time was 62 months (range 1–135 months). A majority of patients in both sets were male. In the total patient cohort, 113 (31%) patients presented with vascular invasion, 88 (24%) had perineural invasion, and 34 (9%) had histologically positive resection margins. The pathological stage of the patients was determined according to the AJCC 8^th^ edition TNM system. A total of 259 (71%) patients had muscle invasion (T2–T4), 88 (24%) had lymph node metastasis (N1–N3), and none had distant metastasis (M1).

### Development and Validation of the Immunohistochemical Signature

**Figure 1:**
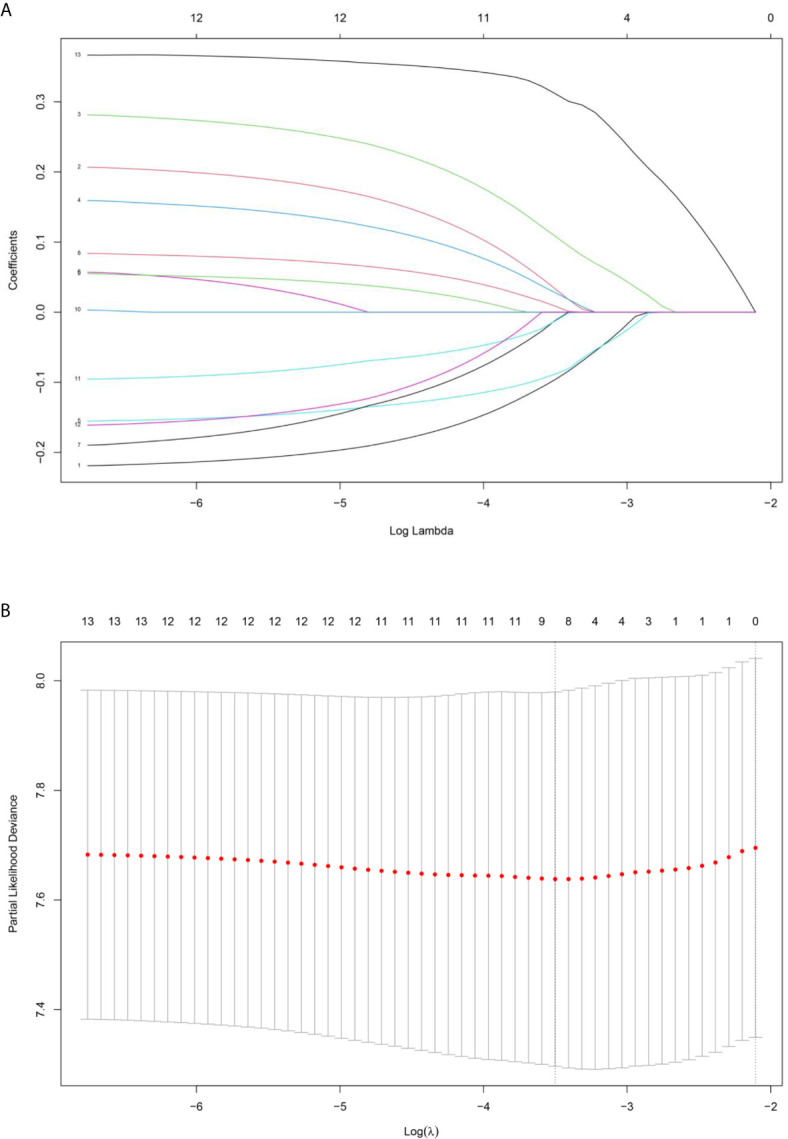
**(A)** LASSO coefficient profiles of the selected IHC markers. **(B)** The tuning parameter (λ) selection used 10‐fold cross‐validation *via* minimum criteria. Partial likelihood deviance was plotted versus log(λ).

**Figure 2:**
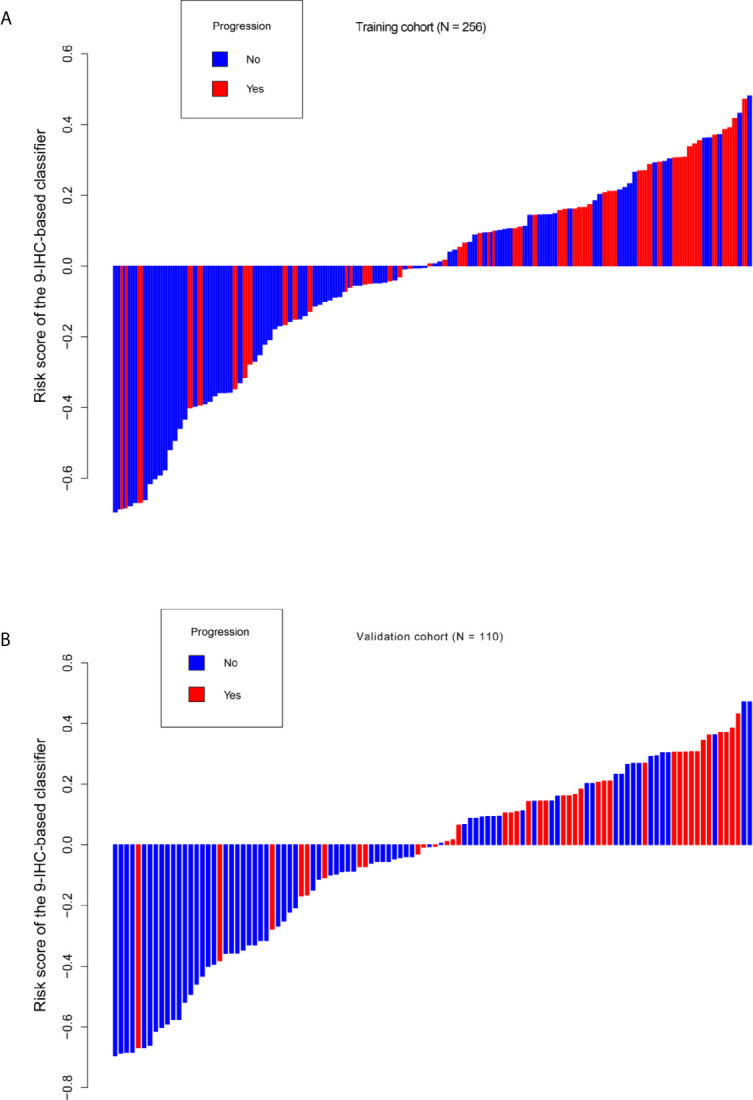
Distribution of risk score based on 9-IHC-based classifier. **(A)** Training cohort. **(B)** Validation cohort.

**Figure 3:**
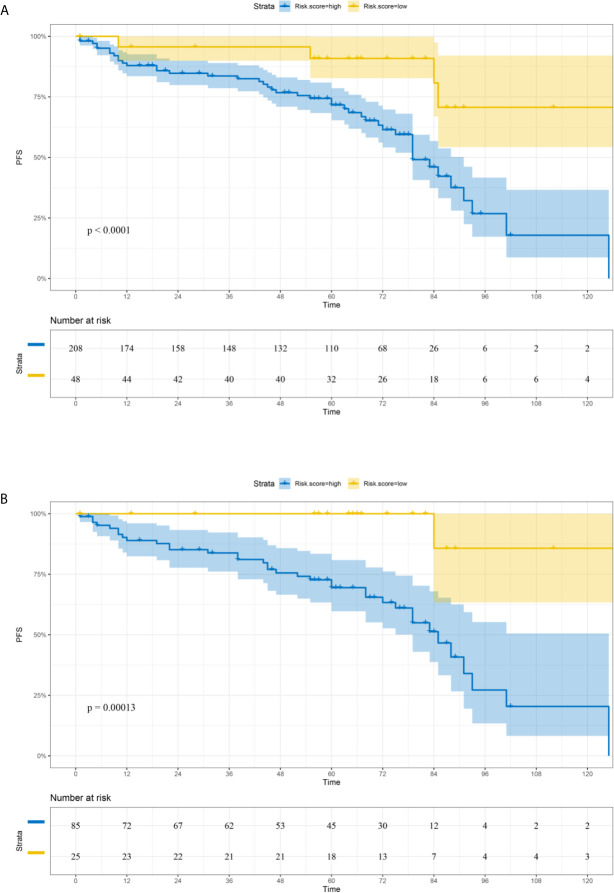
Comparison of DFS in low-risk and high-risk groups stratified by IHC signature. **(A)** Training cohort. **(B)** Validation cohort.

Based on the LASSO Cox regression model, nine prognostic markers (HER2, EGFR, VEGF, CyclinD1, BAX, MDR, p53, p27, and TOPOII) were identified in the training cohort. The risk score of individual patients was calculated according to the expression of these nine IHC markers and their corresponding coefficients (**Figure 1**). Risk score = (0.03338335* HER2) + (0.108497374* EGFR) + (0.027900778* VEGF) + (0.008648065* CyclinD1) + (–0.088330675* BAX) + (–0.0126913* MDR) + (–0.096131019* p53) + (–0.011932695* p27) + (0.311672511* TOPOII). In this formula, the positive marker status equals 1 and the negative status equals 0. The “maxstat” package was used to determine the cut-off value of the classifier, where the risk score ≤0.04311026 represented low risk and the risk score >0.04311026 represented high risk. Furthermore, an adjusted value (–0.04311026) was added to the final formula to simplify the clinical application (**Figure 2**). Based on this IHC prognostic model, 208 (81%) patients were stratified into the low-risk group and 48 (19%) were stratified into the high-risk group in the training cohort. Patients with higher risk scores had poorer outcomes, with 5-year and 10-year DFS of 72% and 18%, respectively, compared with patients with lower risk scores (5-year survival probability: 91%; 10-year survival probability: 71%) **(**p < 0.001, **Figure 3A**). The prognostic value of this nine-IHC prognostic model was further examined in the validation cohort. Further, 85 (77%) patients were classified as low risk and 25 (23%) as high risk; the 10-year survival probability was significantly better in low-risk patients (86%) compared with high-risk patients (20%) (p < 0.001, **Figure 3B**).

### Development and Internal Validation of the Nomogram

**Figure 4:**
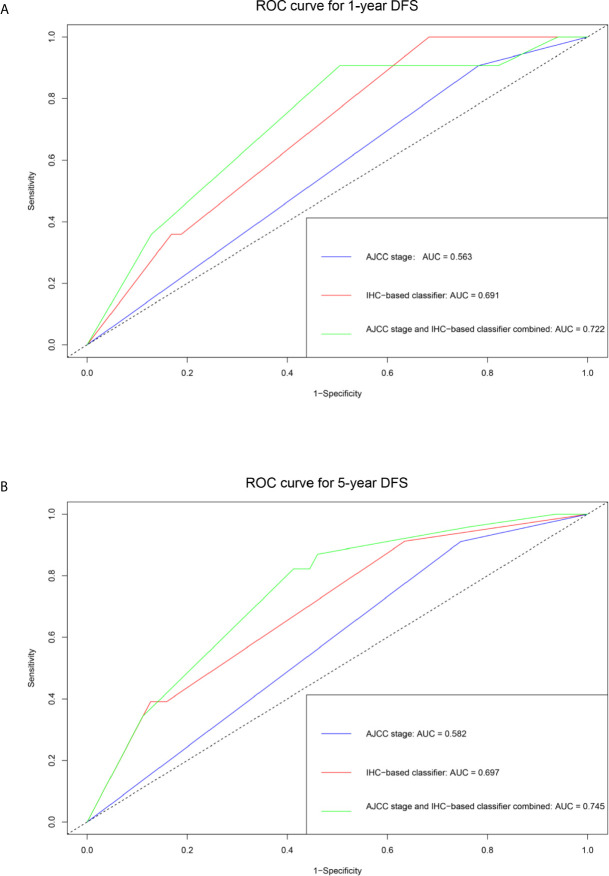
ROC curve analyses of the prognostic value of IHC-based classifier and AJCC stage. **(A)** 1-year DFS. **(B)** 5-year DFS.

**Figure 5:**
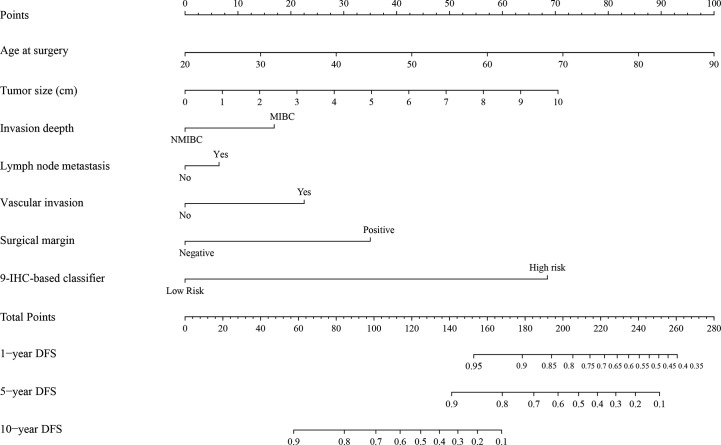
The nomogram for predicting 1-, 5-, and 10-year DFS of bladder cancer patients receiving RC.

**Figure 6:**
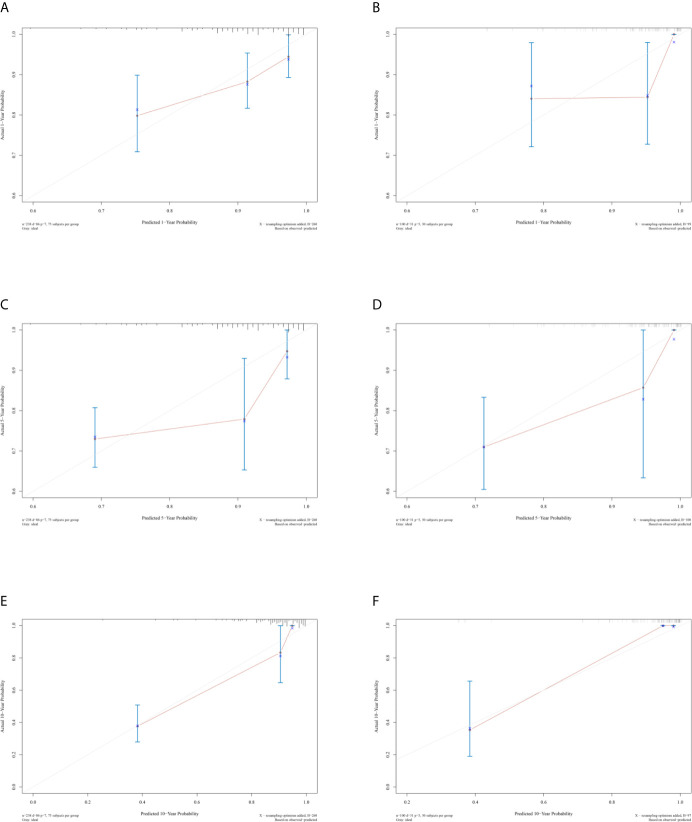
Calibration curves of **(A, C,** and **E)** 1-, 5-, and 10-year DFS for training cohort. **(B, D,** and **F)** 1-, 5-, and 10-year DFS for validation cohort.

The unadjusted and adjusted Cox regression model analysis showed that older age, advanced T stage, lymph node metastasis, high grade, larger tumor size, vascular invasion, resection margin, and high-risk group based on the classifier were identified as independent risk factors associated with poorer DFS (**Table 2**). Notably, the 1-year AUC of the classifier based on the time-dependent ROC curve analysis was 0.691, better than that according to the AJCC 8th edition TNM system (AUC = 0.563). Moreover, the combination of the classifier and the AJCC-based prediction model had the best prediction accuracy (AUC = 0.722) (**Figure 4A**). The 5-year ROC curve analysis also demonstrated the promising prognostic value of the classifier (**Figure 4B**). A prognostic nomogram was then constructed by integrating the classifier and multiple clinicopathological prognostic factors independently associated with DFS (**Figure 5**). By summing each score of all the selected variables, the 1-, 5-, and 10-year survival probabilities of the individual patients were determined. The C-index of the nomograms to predict DFS was 0.78 (95%CI: 0.65-0.94) and 0.68 (95%CI: 0.60-0.77) for the IHC-classifier-based prediction model and AJCC-based prediction model, respectively. The internal and external calibration curves for 1-, 5-, and 10-year DFS also showed high consistency between the estimates using the IHC-classifier-based nomogram and the actual survival probabilities in the training and validation cohorts (**Figure 6**).

**Table 2:** Unadjusted and adjusted Cox regression analyses of DFS in bladder cancer patients.

Covariates	Unadjusted analysis		Adjusted analysis	
	HR (95%CI)	P value	HR (95%CI)	P value
Age at surgery	**1.05 (1.02-1.07)**	<0.001^*^	**1.04 (1.02-1.07)**	0.001^*^
Sex (male *vs.* female)	1.29 (0.72-3.09)	0.286		
Invasion depth (NMIBC vs. MIBC)	**0.42 (0.24-0.73)**	0.002^*^	**0.71 (0.54-0.93)**	0.015^*^
Lymph node metastasis (N1 *vs.* N0)	**2.89 (1.81-4.61)**	<0.001^*^	**1.89 (1.48-2.41)**	0.004^*^
Grade (low grade *vs.* high grade)	**0.11 (0.03-0.44)**	0.002^*^	**0.30 (0.12-0.78)**	0.011^*^
Vascular invasion (present *vs.* absent)	**2.80 (1.80-4.36)**	<0.001^*^	**1.99 (1.14-3.47)**	0.015^*^
Perineural invasion (present *vs.* absent)	**1.99 (0.23-3.21)**	0.005^*^	1.88 (0.97-3.62)	0.060
Surgical margin status (present *vs.* absent)	**2.66 (1.56-4.51)**	<0.001^*^	**2.34 (1.20-4.56)**	0.013^*^
Tumor size	**1.13 (1.03-1.24)**	0.010^*^	**1.16 (1.01-1.34)**	0.030^*^
9-IHC-based classifier (low risk *vs.* high risk)	**0.23 (0.11-0.48)**	<0.001^*^	**0.20 (0.09-0.45)**	<0.001^*^

HR, hazard ratio; CI, confidence interval; MIBC, muscle-invasive bladder cancer; NMIBC, non-muscle-invasive bladder cancer; IHC, immunohistochemical. Bold values and * indicate p < 0.05.

## Discussion

Bladder cancer is a biologically heterogeneous disease with complicated molecular alterations during cancer progression and metastasis (18). Canonical prognostic characteristics, including the AJCC TNM system and grade have limited ability to predict the survival of patients with bladder cancer. Radical cystectomy is the current standard treatment for MIBC, while the potential benefit of radical cystectomy must be weighed against its risks and impact on the quality of life (7). Despite advances in surgery and intravesical therapy, approximately 70% of patients with NMIBC develop tumor recurrence or progression to MIBC. After radical cystectomy up to 50% of patients with MIBC experience local or systemic relapse and eventual decease (19). The relatively high recurrence and progression rate make bladder cancer one of the most complicated malignancies, incurring a huge treatment cost and imposing a large financial burden on the public. Tumor heterogeneity has posed a tremendous challenge to the management of bladder cancer. The IHC analysis of biomarker expression is complementary to the evaluation of tumor morphology and important in the accurate diagnosis of bladder cancer (8). The markers that constitute the diagnostic signature have a varied range of ascribed functions (4, 18). Growing evidence shows that the diagnostic IHC markers can also identify patients at high risk of progression after surgery and improve the disease management of patients in various cancer types (12, 20).

In this study, a nine-IHC signature was constructed using the LASSO Cox regression model analysis for the prediction of DFS in patients with bladder cancer undergoing radical cystectomy. A significant distinction in prognosis was observed between low-risk and high‐risk patients by applying the classifier to the training cohort. Further, the potential value of the signature was validated in the validation cohort, indicating the broad applicability of this classifier. Multivariable Cox regression analyses showed that the high-risk group based on the classifier was an independent risk factor associated with poorer DFS adjusted by clinicopathological characteristics. The time-dependent ROC curve analysis revealed that the IHC signature combined with AJCC staging was a more effective prognostic model than the AJCC staging system alone. Finally, a prognostic nomogram integrating the classifier and multiple clinicopathological prognostic factors was developed for clinical application.

The molecular classification of urothelial bladder cancer has taken significant steps forward in the last decade. In 2012, Sjodahl et al. first identified five major subtypes; urobasal A, genomically unstable, urobasal B, squamous cell carcinoma-like, and infiltrated (21). In 2014, Choi et al. proposed a three-group system; basal, luminal, and p53-like (22). TCGA developed a four-group system at the same time, and in 2017, Robertson et al. updated the TCGA classification system; luminal-papillary, luminal-infiltrated, luminal, neuronal, and basal-squamous (23, 24). Numerous researchers then attempted to develop a reliable IHC panel for predicting patient outcome. The panel components were updated regularly; however, none of them have been recommended by clinical practice guidelines (15, 16).

The LASSO Cox regression model is a popular method for the shrinkage of features and optimal selection of prognostic markers to construct a model. The present study developed a risk-stratification algorithm based on an IHC-based classifier with the purpose to facilitate the clinical management of bladder cancer. Of the 12 biomarkers tested, nine potential predictors were identified. The biological function of biomarkers included in the signature has been previously reported (25). EGFR is a receptor tyrosine kinase involved in the pathogenesis of a variety of cancers. The role of EGFR as a strong independent prognostic marker and therapeutic target in bladder cancer has been well identified (26, 27). HER2 is a transmembrane phosphoglycoprotein belonging to the EGFR family and is known as an established therapeutic target in breast carcinomas (28). HER2 is also overexpressed in a variety of human malignant tumors including bladder cancer. Several studies demonstrated that HER2 overexpression was an independent risk factor associated with unfavorable prognosis in bladder cancer (29). VEGF has been considered as an important factor in pathological angiogenesis, and the VEGF level has been identified as a significant predictor of OS and CSS in patients with bladder cancer (30). CyclinD1 is a regulatory protein in the G1/S transition and its aberrant expression can lead to uncontrolled cell proliferation (31). BAX is an important apoptosis-related molecule that promotes cell apoptosis. Previous studies demonstrated that the expression level of BAX provided the prognostic information of patients with bladder cancer (32). MDR proteins are frequently expressed in untreated bladder cancer and confer resistance to a distinct spectrum of drugs (33). The tumor suppressor gene TP53 is the most commonly mutated gene in human cancer; the mutations of TP53 result in increased p53 nuclear accumulation. The prognostic value of p53 to determine the risk of bladder cancer recurrence and progression has been assessed (34). p27 is a negative cell cycle regulatory gene that potentiates cell cycle arrest in the G1 phase. A decreased p27 protein level has been proved to be associated with the poor prognosis of patients with bladder cancer (35). TOPOII is a DNA gyrase isoform essential in cell cycle. Previous studies have shown its diagnostic value in bladder cancer (36). A particular patient belonging to the high-risk group has more abnormally-activated signaling pathways that promote bladder cancer to relapse and metastasize, eventually resulting in poorer DFS.

In the present study, multiple IHC markers were integrated into one panel based on the LASSO Cox regression model. This multi-marker panel had significantly higher prognostic accuracy compared with any single marker alone (37). All these markers could be easily assessed by IHC based on routine pathological specimens, making the panel a widely available and cost-effective tool to accurately predict the prognosis of patients with bladder cancer. Moreover, a prognostic nomogram integrating the classifier and multiple clinicopathological prognostic factors was also constructed for clinical application. By summing each score of all the selected variables, the 1-, 5-, and 10-year DFS values of the individual patients were determined. The reliability and accuracy of the nomogram allowed personalized disease management of bladder cancer and might eventually enter routine clinical practice.

Despite the promising predictive accuracy of the nine-IHC marker-based nomogram, this study had several limitations derived from its retrospective nature. First, the absence of details with regard to neoadjuvant chemotherapy regimen limited the present analysis. Neoadjuvant chemotherapy has been proved to be associated with improved OS and DFS (7). Second, the presence of carcinoma *in situ* (CIS) has been reported as an independent risk factor associated with poorer RFS (37). However, only four (0.6%) patients present with CIS in the overall patient cohort, and therefore, the prognostic analysis of CIS is not available. Moreover, this signature was validated only in an individual cancer center, and thus further validation from multiple centers and across different populations is expected.

In summary, this study generated and validated a nine-IHC-based classifier for prognostic prediction in patients with bladder cancer undergoing radical cystectomy. A prognostic nomogram was also constructed by integrating the classifier and clinicopathological characteristics. Predicting outcomes of patients with accurate prognostic models can eventually guide the clinical decision making regarding treatment strategy and follow-up scheduling of bladder cancer.

## Data Availability Statement

The original contributions presented in the study are included in the article/**Supplementary Material**, further inquiries can be directed to the corresponding author/s.

## Ethics Statement

The studies involving human participants were reviewed and approved by The institutional ethics committee of Fudan University Shanghai Cancer Center. The patients/participants provided their written informed consent to participate in this study. Written informed consent was obtained from the individual(s) for the publication of any potentially identifiable images or data included in this article.

## Author Contributions

JW conceived the idea and was a major contributor in manuscript writing. Y-CW conducted the data collection and was involved in the manuscript writing. J-MW performed all statistical analyses. W-JL, Q-FW, and HL designed and performed the experiments. BD, D-WY, H-CS, and Y-PZ critically revised the manuscript. All authors contributed to the article and approved the submitted version.

## Funding

This work was supported by the National Natural Science Foundation of China (Project 81772706).

## Conflict of Interest

The authors declare that the research was conducted in the absence of any commercial or financial relationships that could be construed as a potential conflict of interest.
