# Supplementary material for: Prognostic Value of an Immunohistochemical Signature in Patients With Bladder Cancer Undergoing Radical Cystectomy
Source: Front Oncol. 2021 Mar 25;11:641385. doi: 10.3389/fonc.2021.641385 (PMC8027317; doi:10.3389/fonc.2021.641385)
Supplement: Supplementary file 1 [file Table_1.pdf]

Supplementary Table 1 Antibodies information

Antibodies	Details
HER2	recombinant rabbit monoclonal antibody; clone 4B5, Ventana Medical Systems, Inc., Tucson, AZ, USA
EGFR	recombinant rabbit monoclonal antibody; clone 5B7, Ventana Medical Systems, Inc., Tucson, AZ, USA
VEGF	recombinant rabbit monoclonal antibody; clone RB-9031, Subsidiary of Thermo Fisher Scientific, Runcorn, UK
CyclinD1	recombinant rabbit monoclonal antibody; clone SP4-R, Ventana Medical Systems, Inc., Tucson, AZ, USA
BAX	recombinant mouse monoclonal antibody; clone G3-31, Shanghai Long Island Antibody Diagnostica, Inc., Shanghai, China
BCL2	recombinant rabbit monoclonal antibody; clone SP66, Ventana Medical Systems, Inc., Tucson, AZ, USA
Ki67	recombinant rabbit monoclonal antibody; clone 30-9, Ventana Medical Systems, Inc., Tucson, AZ, USA
MDR	recombinant mouse monoclonal antibody; clone P170, Shanghai Long Island Antibody Diagnostica, Inc., Shanghai, China
p53	recombinant mouse monoclonal antibody; clone DO-7, Leica Biosystems Newcastle, Ltd., Newcastle, UK
p27	recombinant mouse monoclonal antibody; clone DCS-72.F6, Shanghai Long Island Antibody Diagnostica, Inc., Shanghai, China
GSTπ	recombinant mouse monoclonal antibody; clone 353-10, Shanghai Long Island Antibody Diagnostica, Inc., Shanghai, China
TOPOII	recombinant mouse monoclonal antibody; clone 3F6, Leica Biosystems Newcastle, Ltd., Newcastle, UK
